# Safety Net kidney after liver transplantation: unexpected renal recovery in a liver transplant recipient after more than 10 months on maintenance hemodialysis – a case report

**DOI:** 10.3389/fneph.2026.1762265

**Published:** 2026-03-19

**Authors:** Phuong-Thu T. Pham, Phuong-Chi T. Pham

**Affiliations:** 1 Kidney Transplant Program, Nephrology Division, Department of Medicine, David Geffen School of Medicine at University of California at Los Angeles (UCLA), Los Angeles, CA, United States; 2 Nephrology Division, Department of Medicine, Olive-View University of California at Los Angeles (UCLA) Medical Center, Sylmar, CA, United States

**Keywords:** acute tubular necrosis, hemodialysis, hepatorenal syndrome, continuous kidney replacement therapy, liver transplantation, continuous renal replacement therapy, safety net, simultaneous liver-kidney transplantation

## Abstract

The incidence and likelihood of kidney function recovery in liver transplant recipients with acute kidney injury (AKI) due to hepatorenal syndrome (HRS) or acute tubular necrosis (ATN) requiring maintenance dialysis remain undefined. Nonetheless, it has been suggested that dependence on dialysis for more than 6 months diminishes the likelihood of renal function recovery. We report a liver transplant recipient with pre-transplantation AKI attributed to HRS with or without ATN who recovered kidney function after more than 10 months on dialysis.

## Introduction

After a successful liver transplant (LT), renal function recovery from presumed hepatorenal syndrome (HRS) with or without acute tubular necrosis (ATN) varies widely among dialysis-dependent patients. One retrospective study involving 834 LT recipients who survived 6 months after transplantation showed that among patients with HRS, those requiring dialysis in the first 3 months post-transplantation had the greatest risk for chronic kidney disease (defined as sustained serum creatinine level > 2.5 mg/dL) and end-stage kidney disease (1). Historically, different programs set forth different dialysis duration thresholds for simultaneous liver kidney transplantation (SLKT), ranging from 4 to 12 weeks, whereas the American consensus guidelines suggested 8 weeks on dialysis as a threshold for SLKT in LT candidates with AKI requiring continuous kidney replacement therapy (formerly known as continuous renal replacement therapy or CRRT) (2–5). Because of the unpredictability of renal recovery among patients receiving dialysis support for presumed HRS with or without ATN, clinicians often felt pressured to perform SLKT when a liver-alone transplant may have sufficed. In 2017, the United Network for Organ Sharing/Organ Procurement and Transplantation Network (UNOS/OPTN) implemented the Safety Net policy, whereby recipients of liver-alone transplant whose renal function does not recover between 60 and 365 days after a successful liver transplant are given increased priority to receive a deceased donor kidney (6). We herein present a case of a liver transplant recipient with pre-transplant AKI requiring dialysis (AKI-D) who recovered renal function after more than 10 months on dialysis while being evaluated for a kidney transplant via the Safety Net. If the patient had presented earlier, she would have likely already received an unnecessary kidney transplant.

## Case report

The patient is a 46-year-old woman with hypothyroidism, morbid obesity, perforated duodenal ulcer requiring surgical repair with gastroduodenostomy, and cirrhosis who was initially admitted to a local hospital for worsening jaundice and bilateral lower extremity edema. Laboratory studies showed serum sodium 133 mmol/L, serum creatinine 1.85 mg/dL (baseline 0.67 mg/dL), INR 2.0, total bilirubin 26.5 mg/dL (10.7 mg/dL three weeks prior), and ammonia 71 µmol/L. Despite full medical support, including albumin, midodrine, and octreotide for presumed HRS, her renal function and hemodynamic status continued to decline (peak serum creatinine 4.67 mg/dL), necessitating continuous kidney replacement therapy (CKRT). At this point, the patient was transferred to our facility for LT evaluation.

At transfer, the patient had a MELD-Na score of 39 (up from 24 three weeks prior). Vital signs were blood pressure 120/55 mmHg (on pressure support), pulse rate 69 bpm, temperature 96.6°F, respiratory rate 11/min, and body mass index 53.5 kg/m^2^. Medications included levothyroxine, lactulose, midodrine, and rifaximin. Physical examination was remarkable for icteric conjunctiva, jaundice, ascites, anasarca, and asterixis. There was no history of alcohol use. Viral hepatitis panel, autoimmune hepatitis serologies, hemochromatosis, and alpha-1 antitrypsin deficiency were negative. Non-contrast CT of the abdomen/pelvis showed cirrhosis, mild splenomegaly, and moderate ascites. Bilateral kidneys and ureters were unremarkable.

Given her morbid obesity, a diagnosis of non-alcoholic steatohepatitis (NASH) liver cirrhosis was made (currently known as metabolic dysfunction-associated steatohepatitis or MASH). She remained on CKRT (initiated 1 week prior to transfer) for oliguric AKI presumably precipitated by HRS and/or ATN. After an extensive medical and surgical evaluation and recent AKI requiring CKRT of less than 6 weeks duration, the patient was listed as a candidate for isolated liver transplantation and was offered a liver transplant after 14 days. She received basiliximab induction and standard maintenance triple immunosuppressive therapy with tacrolimus, mycophenolate mofetil, and prednisone. Her post-operative course was uneventful. However, she remained oliguric with less than 150 mL of urine output daily, requiring continuation of post-transplant kidney replacement therapy (KRT). She was discharged on hospital day 37 with outpatient maintenance hemodialysis three times a week.

**Figure 1:**
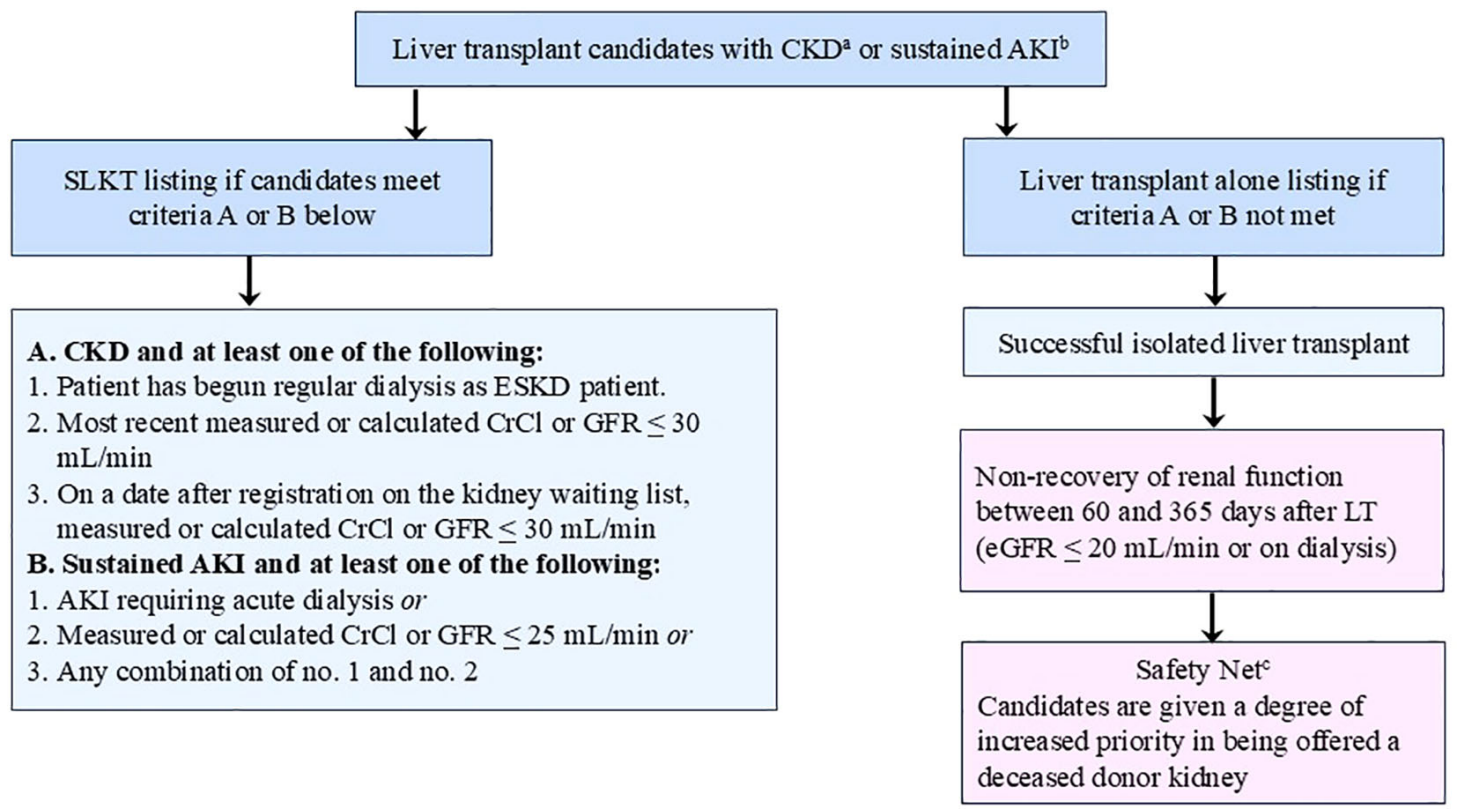
UNOS/OPTN medical eligibility criteria for SLKT listing and safety net policy ^a^CKD defined as measured or calculated CrCl or GFR of ≤ 60 mL/min for greater than 90 consecutive days prior to listing; ^b^Sustained AKI defined as AKI present for at least 6 consecutive weeks (must be documented in patient’s medical records every 7 days); ^c^Patient is eligible for kidney after liver transplantation listing via Safety Net in the setting of non-recovery of renal function between 60 and 365 days after LT. UNOS/OPTN, United Network for Organ Sharing/Organ Procurement and Transplantation Network; SLKT, simultaneous liver kidney transplantation; CKD, chronic kidney disease; AKI, acute kidney injury; ESKD, end-stage kidney disease; CrCl, creatinine clearance; GRF, glomerular filtration rate; LT, liver transplantation.

**Figure 2:**
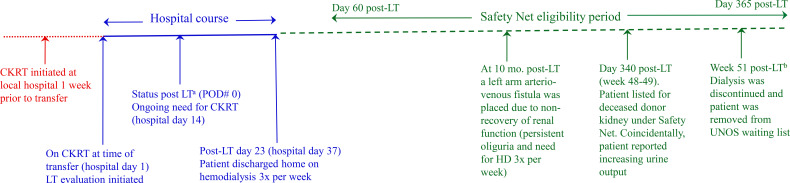
Patient’s clinical course depicting sequence of isolated liver transplantation, dialysis duration, safety net listing, renal recovery, and removal from UNOS kidney waiting list. ^a^Patient received isolated liver transplantation because she had not yet met UNOS criteria for SLKT at the time of listing for liver transplantation (SLKT eligibility criteria defined in **Figure 1**); ^a^At week 51 post-LT, 24h urine studies showed CrCl 34 ml/min/1.73m^2^. At 5-year follow-up: SCr 1.26-1.48 mg/dL (eGFR 42–51 ml/min by CKD-EPI 2021). LT, liver transplantation; CKRT, continuous kidney replacement therapy; POD, post-operative day; HD, hemodialysis; CrCl, creatinine clearance; UNOS, United Network for Oragn Sharing; AVF, arteriovenous fistula; SCr, serum creatinine; eGFR, estimated glomerular filtration rate; CKD-EPI, Chronic Kidney Disease Epidemiology Collaborative.

Ten months later, her Permacath was converted to a left arm arteriovenous fistula due to ongoing non-recovery of renal function (persistent oliguria with pre-dialysis serum creatinine 4–5 mg/dL). She was referred to our center for kidney transplantation evaluation and was subsequently placed on the United Network for Organ Sharing (UNOS) waiting list 340 days post-LT under the Safety Net policy. Coincidentally, at this time, the patient reported improved urine output to approximately “half of normal” volume (not quantified). Her urine output gradually improved and allowed for reduced ultrafiltration requirements. Serum creatinine after holding one session of dialysis was 2.4 mg/dL (eGFR of 23 ml/min/1.73m^2^, CKD-EPI 2021). Concomitant 24-hour urine collection revealed a volume of 925 ml, 1.2 g creatinine, and creatinine clearance of 34 mL/min. Microalbumin/creatinine ratio was 48 mcg/mg creatinine. Dialysis was discontinued, and the patient was removed from the UNOS waiting list. Her renal function improved to 2.11 mg/dL one month after dialysis discontinuation and gradually reached a nadir of 1.25 mg/dL (eGFR 50 mL/min/1.73m^2^) over the ensuing 6 months. At 5-year follow-up, her serum creatinine fluctuated between 1.26-1.48 mg/dL, with eGFR 42–51 mL/min/1.73m^2^ without microalbuminuria. The UNOS Safety Net eligibility criteria and the patient’s clinical course are summarized in **Figures 1** and **2**, respectively.

## Discussion

With the introduction of the model for end-stage liver disease (MELD) score for the allocation of liver transplantation in February 2002, a striking 278% increase in the number of simultaneous liver-kidney transplantations (SLKT) performed was observed in the 9-year post-MELD vs. the preceding 9-year pre-MELD era (n=2914 vs. n=1049 in the 9-year post-MELD vs. the preceding 9-year pre-MELD era, respectively) (3). Because the MELD score-based allocation of liver transplantation prioritizes patients with renal dysfunction, concerns arose regarding inappropriate kidney allocation to those with potentially reversible AKI.

In August 2017, the UNOS/OPTN committee set forth well-defined medical eligibility criteria for SLKT listing to assure kidney allocation only to those truly in need (**Figure 1**) (6). While these criteria serve to limit SLK recipients to patients with AKI without likelihood of renal recovery, the policy recognizes that a subset of patients may benefit from SLKT but are offered a liver alone because they do not meet criteria for simultaneous organ transplants at the time of listing for a liver transplant (or because of peri-operative medical instability after liver transplantation or poor kidney organ quality). To capture these patients and address their needs, the UNOS/OPTN committee concurrently implemented the Safety Net policy, whereby recipients of liver-alone transplant with non-recovery of renal function between 60 and 365 days after a successful liver transplant are given a certain degree of priority in being offered a deceased donor kidney (non-recovery defined as GFR remains ≤ 20 mL/min or dialysis dependence) (6) (**Figure 1**).

Our current patient received isolated liver transplantation in the setting of AKI requiring dialysis (AKI-D) of less than 6-week duration. She was subsequently listed for a kidney transplant 340 days post-liver transplantation (post-LT) under the Safety Net policy because of non-recovery of renal function. However, her kidneys recovered adequate function, permitting dialysis discontinuation. To our knowledge, renal recovery after more than 10 months on maintenance dialysis in liver transplant recipients has not been reported.

In one single-center retrospective study consisting of 126 recipients of living donor liver transplant, post-LT renal recovery occurred in 69.0% of patients with HRS (defined as the return of serum creatinine to ≤1.5 mg/dl and the absence of persistent need for kidney replacement therapy). The percentage of patients with pre-LT AKI requiring kidney replacement therapy (CKRT or hemodialysis) was significantly higher in the non-renal recovery compared with renal recovery groups (non-renal recovery vs. recovery: 48.7% vs. 25.3%, respectively, p=0.01), whereas the percentage of patients with post-LT AKI requiring kidney replacement therapy was comparable between the two groups (non-renal recovery vs. recovery: 84.6% vs. 73.6%, respectively, p=0.25). Notably, the time interval from HRS diagnosis to LT of ≥ 38 days had a negative impact on post-LT renal recovery (p = 0.01) (7). The time to renal recovery was not reported.

Retrospective studies in the non-transplant setting demonstrated that of 1,968,253 adults and children who were started on dialysis in the outpatient setting, 4.3% of adults (83,302/1,953,881) and 3.8% of children (547/14,372) recovered kidney function within 1 year (defined as survival and discontinuation of dialysis for ≥90-day period). Among those who recovered, the median time to recovery was 73 days (interquartile range [IQR] 43-131) in adults and 100 days (IQR 56-189) in children. The diagnoses associated with the highest recovery rates of recovery were ATN and acute interstitial nephritis in both adults and children, where 25%-40% of patients recovered kidney function (8). Similar studies in the transplant setting are lacking.

The implementation of uniform criteria for SLKT listing and Safety Net policy reduced the number of SLKT performed in the 3-year post- compared with the 3-year pre-policy period (n=1702 vs. 1737, respectively) (9). Although policy change seemingly effectively resulted in an absolute decrease in the number of SLK transplants, the UNOS/OPTN database showed a concurrent greater absolute increase in the number of Safety Net kidney after liver (KAL) transplants in the immediate 2.5 years after policy change (between 8/10/17-2/9/20, 132 KAL transplants were performed via Safety Net with an average waiting time of 57 days from the time of listing) (9). The greater number of KAL transplants following implementation of the Safety Net policy raises the possibility that the Safety Net eligibility criteria for KAL transplant may be too lenient.

Notably, at the authors’ institution, between August 2017 and September 2022, 3 of 59 Safety Net patients had significant renal function recovery and were removed from the UNOS kidney waiting list (10). Of the three patients whose renal function recovered, one was on dialysis for 4 months, one for 6.5 months, and the third for 10 months (current case report/unpublished data). Of the 59 Safety Net patients, 40 (67.8%) received a kidney transplant at a mean of 341 ± 393 days (range 2–1624 days) from the time of kidney listing to transplantation (median 177 days; interquartile range 17–621 days) (10). Had our current patient presented at 60 days or at any time before 340 days, when her renal function recovered, she would have likely received an unnecessary kidney transplant.

Our case report is not without limitations, given the lack of a kidney biopsy. The diagnosis of HRS-AKI was made in the context of acute liver dysfunction, normal kidneys on CT imaging, absence of shock, and no history of nephrotoxic medication use. Urine studies could not be reliably analyzed or interpreted because the patient was oliguric and had an indwelling Foley catheter at the time of transfer. Whether the current patient had HRS-AKI or ATN, or both was speculative. Nonetheless, our current case and review of our own center’s experience highlight the potential for renal recovery after more than 60 days on dialysis among LTA recipients with ongoing AKI-D presumed to be secondary to HRS and/or ATN. In one single-center retrospective study, 1- and 5-year patient survival and renal function after LT were significantly worse for those with ATN compared with their HRS counterparts (11).

Potential mechanisms underlying late renal function recovery after AKI-D remain to be elucidated but may include delayed hemodynamic normalization, microvascular remodeling, resolution of systemic inflammation, or prolonged tubular repair, the latter potentially exacerbated by calcineurin inhibitor (tacrolimus) nephrotoxicity. Further studies are needed. Repeated episodes of intradialytic hypotension can delay the renal tubular regeneration process due to its impact on cellular ATP depletion and direct injury to tubular epithelial cells. A randomized trial in the non-transplant setting demonstrated that in patients with AKI-D, a conservative dialysis strategy (in which hemodialysis or isolated ultrafiltration was performed only when specific metabolic or clinical indications were met) resulted in a shorter time to and higher rates of kidney recovery compared with conventional thrice weekly dialysis (64% vs 50%, p=0.04 in unadjusted analysis). Dialysis-associated hypotension occurred less frequently in the conservative group. In essence, dialysis per se may have delayed recovery from AKI (12).

## Conclusion

To our knowledge, renal recovery after more than 10 months on maintenance dialysis has not been reported in the transplant or non-transplant settings. The incidence and predictors of delayed recovery among liver-alone transplant recipients with ongoing AKI-D remain uncertain.

Although our case is exceptional, studies evaluating the incidence and predictors of renal function recovery after more than 60 days on dialysis among recipients of liver transplant alone under the Safety Net may be invaluable. Such studies may allow clinicians to assess the probability of renal recovery in individual patients as well as determine dialysis duration thresholds for renal function recovery. Judicious use of kidney organs can help avoid unnecessary increased waitlist times and waitlist mortality for ESKD patients in true need of an isolated kidney transplant.

## Data Availability

The original contributions presented in the study are included in the article/supplementary material. Further inquiries can be directed to the corresponding author.
